# Enhancement of Physical Appearance, Skin Permeation, and Odor Reduction Using Liposome of Hydrolyzed Salmon Collagen for Cosmetic Products

**DOI:** 10.1155/2024/7843660

**Published:** 2024-04-24

**Authors:** Thanaporn Amnuaikit, Rajeev Shankar Rajagopal, Krisana Nilsuwan, Soottawat Benjakul

**Affiliations:** ^1^Drug Delivery System Excellence Center, Department of Pharmaceutical Technology, Faculty of Pharmaceutical Sciences, Prince of Songkla University, Hat Yai, Songkhla, Thailand; ^2^International Center of Excellence in Seafood Science and Innovation, Faculty of Agro-Industry, Prince of Songkla University, Hat Yai, Songkhla, Thailand

## Abstract

Hydrolyzed collagen (HC) derived from salmon (*Oncorhynchus nerka*) skin possesses properties that can nourish the skin, and it is one of the active ingredients used in cosmeceutical products for moisturizing the facial skin. However, HC solution gives off a fishy odor and it is gray in color that makes the product unacceptable for cosmetic purposes. This study aimed to use liposome-encapsulated hydrolyzed salmon collagen to improve its physical appearance, skin permeation, and eliminate the fishy odor. Two percent of HC and vitamin B3 (VitB3) were used as active ingredients to incorporate into liposomes. Phosphatidylcholine, cholesterol, and Tween 80 at a suitable weight ratio of 8 : 2 : 1 produced nano-sized vesicles (170.6 ± 0.70 nm) with the highest percentage of entrapment efficiency (95.72 ± 2.00%) of VitB3 and (49.63 ± 1.74%) of HC. Skin permeation and odor detection of the HC-VitB3 liposome were studied using Franz's diffusion cell and gas chromatography, respectively, and compared with HC-VitB3 solution. Subsequently, facial serums were formulated using HC-VitB3 liposomes and HC-VitB3 solutions, and a product satisfaction test was conducted with 100 volunteers to determine their preferred product. The results of the studies of HC-VitB3 liposome serum showed improved formulation appearance, enhanced skin permeation, and better odor elimination compared to the HC-VitB3 serum. Furthermore, seventy-three volunteers in the product satisfaction test preferred and selected the liposomal serum for its superior scent. From all the experimental results, it could be seen that liposomes can help increase skin penetration, and undesirable odors and colors can be masked by the appropriate lipid bilayer structure of liposomes.

## 1. Introduction

Collagen constitutes a substantial group of fibrous proteins located outside the cell, most commonly present in the bodies of both humans and animals. It comprises approximately 30% of all connective tissues in animals and ranks highest in terms of relative organ weight [1]. For instance, it is found in 70–80% of the skin [2]. Collagen is a substance that the body can naturally produce. Throughout our lives, there is a continuous process of synthesizing new collagen to replace the old collagen that has deteriorated. However, this process becomes imbalanced as we age because the degradation of collagen outpaces its production. Marine collagens, which can be obtained from invertebrate marine animals or fish offer several advantages, including a high yield and no risk of disease transmission [3]. Hydrolyzed collagen (HC), particularly the one sourced from fish skin or scales, has garnered attention as a promising source of biologically active peptides. It has also received recognition as a Kosher and Halal product, making it widely accepted [4]. In this study, HC from salmon skin was provided by the International Center of Excellence in Seafood Science and Innovation, Faculty of Agro-Industry, Prince of Songkla University, Thailand. Fat-free HC powder was produced through the optimization of a one-step hydrolysis process using mixed proteases (papain and alcalase) [4]. The molecular weight (MW) distribution of defatted HC from salmon skin, representing proteins or peptides was in the range of 102–10,175 Da [5]. Moreover, the HC powder exhibited a reduced fishy odor and a decrease in lipid oxidation products, making it a potential candidate for use in cosmetic products [4, 5]. However, despite the solubilization of the defatted HC powder in water to form a solution, a persistent fishy odor remained, and even intensified over an extended period. Therefore, the utilization of liposomes to encapsulate hydrolyzed salmon collagen offers an innovative and promising approach to enhance the quality of cosmetic products. Furthermore, to formulate an effective, nourishing, and synergistic cosmetic product, vitamin B3 (VitB3) or nicotinamide was utilized as one of the active ingredients in liposome serum. VitB3 plays a crucial role in enhancing collagen production, regulating the excessive deposition of glycosaminoglycans, and preventing protein glycation. Glycation can lead to the crosslinking of collagen and elastin molecules, resulting in skin stiffness and rigidity, consequently altering the skin's viscoelastic properties [6]. Moreover, VitB3 contributes to improved skin radiance, thereby expanding the potential for developing a successful and beneficial product within the cosmetic market.

Liposomes are small particles ranging from nanometers to micrometers in size. They appear as spherical vesicles containing natural or synthetic phospholipids, organized in a double-layered structure known as a lipid bilayer. This molecular structure comprises both a polar (hydrophilic) part and a nonpolar (hydrophobic) part. This structural arrangement enables liposomes to transport substances that are polar, nonpolar, or amphiphilic. The distribution of these substances within the liposome vesicles depends on their solubility and hydrophobicity. Soluble molecules are typically located within the central cavity of the liposome, while hydrophobic molecules are located within the carbon chain of lipid bilayer [7]. Liposomes can aid in diminishing the undesired odor associated with various drugs, substances, or extracts. A former study utilized fish oil as an ingredient in bread production prepared in the form of liposomes to reduce the fish oil odor [8]. Furthermore, liposomes can assist in masking the color of various substances which are encapsulated within the vesicles [9, 10]. Liposomes are even used for textile and engineering applications, where moringa oil [11] and limonene [12] are transferred to mask using impregnation method. These processes could alleviate the discomfort caused to the skin by the masks and maintain a pleasant scent of limonene within the masks. Numerous studies have explored the permeability and irritation-reducing properties of liposomes [13–15]. Liposomes operate with a mechanism that facilitates the deeper penetration of substances into the skin while preventing small molecules from entering the circulatory system. In the context of minimizing skin irritation, liposomes can decrease the chance of such reactions. This is because the substances are stored within the liposomes, preventing direct contact with the skin and consequently averting irritation.

The development of HC-VitB3 serum using liposomes is promising and provides better user appeal, as it effectively reduces the fishy odor of collagen, enhances its color, improves permeability, and reduces skin irritation. This research involves a comparative study between the newly developed HC-VitB3 liposome serum and conventional HC-VitB3 serum formulations, covering various aspects such as odor, color, skin permeability, and product satisfaction as evaluated by volunteers.

## 2. Materials

Hydrolyzed salmon collagen (MW 102–10175 Da) was provided by the International Center of Excellence in Seafood Science and Innovation, Faculty of Agro-Industry, Prince of Songkla University, Thailand. Soybean phosphatidylcholine (SPC) and cholesterol (CHOL) were purchased from Sigma-Aldrich (Missouri, USA). Nicotinamide (VitB3), sodium carboxymethyl cellulose (SCMC, 1500–2500 cps), disodium ethylene diamine tetra acetate (EDTA), and propylene glycol were purchased from P. C. Drug Center Co., Ltd. (Bangkok, Thailand). Tween 20, Tween 80, sorbitan monooleate (Span 80), and phenoxyethanol were purchased from Chanjao Longevity Co., Ltd., Bangkok, Thailand. Methanol and ethanol (analytical grade) were purchased from RCI Labscan Limited (Bangkok, Thailand). Water was purified using a Milli-Q system (Millipore, Bedford, MA, USA).

## 3. Methods

### 3.1. Preparation of HC-VitB3 Liposomes

The HC-VitB3 liposome was prepared using a modified ethanol injection method [14]. To briefly describe the process, each material was carefully weighed and placed into two separate containers: one for the water phase and another for the lipid phase, with ethanol serving as the solvent. The temperature of both phases was maintained at 60°C before mixing them together. Subsequently, the ethanol was removed using a rotary evaporator at 50°C to form the liposome. HC and VitB3 were fixed at 2.0% (w/v) in the formulations. CHOL, SPC, and water were the primary components of the liposomes [16]. The concentrations and ratios of all components are shown in Table 1.

### 3.2. Characterization of HC-VitB3 Liposomes

#### 3.2.1. Physical Properties of HC-VitB3 Liposomes

HC-VitB3 liposomes were evaluated for color and appearance through visual assessment. In addition, a smelling test for the formulations was conducted by a formulator with permission from the Ethics Committee of the Faculty of Pharmaceutical Sciences at Prince of Songkla University, Thailand, which approved this study before its execution. The assessment involved grading on a scale of five levels represented by plus symbols (+ to +++++), and comparisons were made against a HC-VitB3 solution. The screening results of physical properties were used to select HC-VitB3 liposomes for further investigation. The selected formulations had their color determined using a CM 700d spectrophotometer (Konica Minolta, Inc., Tokyo, Japan). Color parameters (CIELAB) *L*^*∗*^(0 = black, 100 = white), *a*^*∗*^(+ value = red, −value = green), and *b*^*∗*^ (+ value = yellow, −value = blue) were determined for studying the color of formulations. Total difference in color (Δ*E*^*∗*^) between the HC-VitB3 liposome formulation and the HC-VitB3 solution was calculated using the following equation [4]:

(1) $$\begin{array}{c} ∆E^{*}=\sqrt{{{∆L}^{*}}^{2}+{{∆a}^{*}}^{2}+∆{b^{*}}^{2}}, \end{array}$$

where Δ*L*^*∗*^, Δ*a*^*∗*^, and Δ*b*^*∗*^ are the differences between the corresponding color parameter of the HC-VitB3 liposome and that of HC-VitB3 solution.

#### 3.2.2. Determination of Vesicle Size, Size Distribution, and Zeta Potential

The vesicle size, size distribution, and zeta potential of the formulations were assessed at 25°C using a zeta potential analyzer (Model ZetaPALS, Brookhaven Instrument, Corp, New York, USA). For each measurement, 10 *μ*l of the samples were dispersed in 4 ml of Milli-Q water. All measurements were conducted in triplicate and are reported as mean ± standard deviation [16].

#### 3.2.3. Determination of Entrapment Efficiency

Two methods were used for the determination of entrapment efficiency in liposomes, based on the characteristics of the active substances. This was due to the presence of two types of active substances: HC and VitB3.

In case of VitB3, each prepared liposome (5 ml) was centrifuged at 24,000 rpm, 4°C for 2 hours using an ultracentrifuge (Optima™L-100XP, Beckman, USA). Then, the supernatant was analyzed using ultraviolet visible (UV) spectroscopy to measure the amount of free VitB3 in the formulations (F). The total amount of VitB3 was measured after breaking the vesicles with triton X-100 at a ratio of 1 : 1 of sample and triton X-100 in the formulation (T). VitB3 content in formulation was measured using a modified UV spectrophotometer method [17]. Five solutions (10–50 *μ*g/ml) of different concentrations were prepared from the standard stock solution of VitB3 for linearity study. The absorbance of these solutions was observed against distilled water as blank at 254 nm, and the obtained data were used for plotting the calibration curve.

In case of HC, encapsulation efficiency was evaluated as the percentage difference between the free HC (F) and the total HC (T). The amount of HC in the solution was determined using a ninhydrin assay, which is widely used for the quantification of amino acids. The procedure was slightly modified based on the previously published protocol by Moore and Stein [18]. In brief, either 20 *μ*l of free HC or total HC phase was mixed with 300 *μ*l of 10% Triton X-100 and vortexed to obtain a homogeneous solution. Then, 50 *μ*l of 2% ninhydrin in absolute ethanol was added, and this mixture was heated for 5 min at 95°C, and cooled down to 25°C. The Ruhemann's purple formed by the reaction was measured at 570 nm. The concentration of HC in the sample was calculated by referring to the HC standard curve. Blank liposome was included as a negative control. The percent entrapment efficiency (%EE) was calculated using the following equation [14]:

(2) $$\begin{array}{c} \%\text{EE}=\frac{\left(T-F\right)}{T}\times 100\%, \end{array}$$

where % EE is the percent entrapment efficiency, *T* is the total amount of VitB3/or HC in the formulation, and *F* is the free VitB3/or HC amount.

#### 3.2.4. Determination of Vesicle Morphology

A scanning electron microscope (SEM) was utilized to examine the morphology of liposome vesicles. Sample preparation for SEM assessment followed the method described by Lujan et al. in 2019 [19]. A 2% w/v solution of osmium tetroxide in phosphate buffer solution (PBS) was used as a negative lipid-targeting stain fixation solution. Subsequently, it was essential to sputter-coat these samples with a 20 nm thick layer of iridium to eliminate electron charging before acquiring SEM images (JSM5800LV, JOEL, Japan).

### 3.3. Analysis of Odorant in HC-VitB3 Liposome

A gas chromatography-tandem mass spectrometer (7890 B GC–7000D MS, Agilent Technologies, USA) with headspace-solid phase microextraction-gas chromatography-electron ionization/mass spectrometry (HS-SPME-GC-EI/MS) technique was used to analyze HC-VitB3 liposome and HC-VitB3 solution odorant. SPME fiber used in this technique was Carboxen®/divinyl benzene (DVB)/polydimethyl siloxane (PDMS) (2 cm), 50/30 *μ*m, Stableflex™ (Agilent Technologies, USA). The gas chromatography conditions were as follows: Agilent 7890 B, column HP-5MS, ultra inert: 30 m × 250 *μ*m × 0.25 *μ*m. The temperature programming was as follows: the initial temperature of 40°C was held for 2 min; then it was increased to 250°C at 10°C/min, which was held for 6 min. The following conditions were applied: transmission line temperature: 280°C; ion source temperature: 210°C; mass scanning range: 33–400 amu; carrier gas He, constant current mode, flow rate: 1.0 ml/min; no split injection was used [20]. The possible compounds were identified by NIST17 library and Wiley 10 and NIST14 libraries with match factor criteria >90%.

### 3.4. Evaluation of HC-VitB3 Liposome on Cell Proliferation, Migration of Human Fibroblast, and Soluble Collagen Production

#### 3.4.1. Cell Culture

*(1) Skin Fibroblast Cell Line*. The human skin fibroblast cell line (BJ, ATCC: CRL-2522, USA, passage No. 5) was cultured in Eagle's Minimum Essential Medium (MEM, Gibco, USA) containing 10% fetal bovine serum (FBS, Gibco®, USA) and antibiotics (100 U penicillin and 100 U/ml streptomycin, Gibco®, USA) under 5% CO_2_ at 37°C. The medium was changed every alternate day. When the cells reached confluence, they were harvested using 0.05% trypsin-EDTA (Gibco®, USA), followed by the addition of fresh culture medium to create a new single cell suspension for further incubation [21, 22].

*(2) Skin Keratinocyte Cell Line*. The human skin keratinocyte cell line (HaCat, CLS no. 300493, Geramany, passage No. 48) was cultured in Dulbecco's Modified Eagle Medium (DMEM, Gibco®, USA) containing 10% fetal bovine serum (FBS, Gibco®, USA) and antibiotics (100 U penicillin and 100 U/ml streptomycin, Gibco®, USA) under 5% CO_2_ at 37°C. The medium was changed every alternate day. When the cells reached confluence, they were harvested using 0.05% trypsin-EDTA (Gibco®, USA), followed by the addition of fresh culture medium to create a new single cell suspension for further incubation [21, 22].

#### 3.4.2. Cell Viability Assay

The human skin fibroblast cell lines (BJ) and human skin keratinocyte cell line (HaCat) at a concentration of 1 × 10^5^ cells/ml were seeded into a 96-well plate in complete media. After 24 h, the samples (blank liposome, HC-VitB3 solution, and HC-VitB3 liposome) of various concentrations (19.5, 39, 78, 156, 312, 625 *μ*g/ml, 1.25, and 2.5 mg/ml) in fresh medium were added into the culture plates, and cells without a sample was served as a control. After incubation for 24 h, 3-(4, 5-dimethylthiazolyl-2-yl)-diphenyl-tetrazolium bromide (MTT) solution (5 mg/ml) assay was performed to evaluate cell activity. In brief, the cells were treated with 80 *μ*l of fresh media along with 20 *μ*l of MTT solution and incubated at 37°C under 5% CO_2_ for 4 h. Thereafter, media containing MTT was removed, and 100 *μ*l of DMSO was added. The absorbance was determined by a microplate reader (Biohit 830, Biohit®, Helsinki, Finland) at a wavelength of 570 nm. The percentage of cell viability was calculated and compared to control [5, 21, 22].

#### 3.4.3. In Vitro Scratch Assay

The spreading and migration capabilities of human skin cells were assessed using a scratch assay (injury to the cell monolayer) that measured the expansion of cell population on a surface. The BJ and HaCat cells line at a concentration of 1 × 10^6^ cells/well in complete media were seeded in a 12 well plate. Once the confluent monolayer was formed, a linear scratch was generated in the monolayer with a sterile pipette tip. Any cellular debris was removed by washing with phosphate buffer saline (PBS) and replaced with 2 ml of samples. The complete media without sample served as a control. Photographs were taken at a 10× magnification using a microphotograph (Olympus CK2, Japan) on day 0, after which the plates were incubated at 37°C with 5% CO_2_, and photographs were taken at days 1 and 2. The images acquired for each sample were further analyzed quantitatively by using computing software (ImageJ/Java). By comparing the images from days 0–2, the distance of each scratch closure was determined and the percentage migration rate was calculated. Average of left scratch and right scratch were taken separately. Percent migration was calculated for left scratch and then right scratch using the following equation:

(3) $$\begin{array}{c} \%\text{Migration rate}=\frac{\text{average distance between scratch}\left(\text{day }0\right)-\text{average distance between scratch}\left(\text{day }1\right)}{\text{average distance between scratch}\left(\text{day }0\right)}\times 100. \end{array}$$

Samples were measured in quadruplicates. Percent rate of migration obtained from all four wells were averaged and recorded [22].

#### 3.4.4. Determination of Soluble Collagen Production

The human skin fibroblast BJ cell line at an initial concentration of 1 × 10^5^ cell/well was seeded in a 96-well plate. After 24 h, the samples of various concentrations in fresh medium were added into the culture plates, respectively. After incubation for 24 h, the supernatants were collected. Then, the total amount of soluble collagens was assayed using the Sorcol® collagen assay kit (Biocolor Ltd., UK). In brief, 100 *μ*l of experimental cell supernatant was mixed with 200 *μ*l of dye solution and mixed gently at room temperature for 30 min. After that the samples were centrifuged at 15, 000g for 10 minutes to form a pellet of collagen. The supernatant was removed and the produced soluble collagen was dissolved by 500 *μ*l of alkali reagent. The resultant solution from alkali reagent was transferred to the 96-well plate and the absorbance was determined by a microplate reader at a wavelength of 540 nm. The amount of collagen was calculated based on a standard curve of soluble collagen [22].

### 3.5. In Vitro Skin Permeation of HC-VitB3 Liposome

Skin permeation study of the selected HC-VitB3 liposome and HC-VitB3 solution was carried out using a modified Franz diffusion cell (Model Hanson 57-6M, California, USA). Pig ear skin that was used as the skin model was provided by a pork butcher (local market in Hat Yai, Thailand); it was mounted between the donor and receptor compartments [16, 23]. HC and VitB3 (2% w/v) liposome and HC-VitB3 solution (2 ml) were placed on the skin covered by the upper compartment of a Franz diffusion cell. The samples (2 ml) were withdrawn at 0.5, 1, 2, 4, 8, 16, and 24 h from the receptor chamber and immediately replaced with an equal volume of fresh receptor medium (PBS pH 7.4). The content of VitB3 in receptor fluid was analyzed by a UV spectrophotometer. Five concentrations of VitB3 solution in PBS (1–5 *μ*g/ml) were prepared for linearity study. The permeation profile was plotted between cumulative amount of VitB3 permeated per unit area and function of time. The cumulative amount (*Q*_*t*_) of VitB3 permeation per unit area was calculated using the following equation:

(4) $$\begin{array}{c} Q_{t}=\frac{\left(C_{n}V+\sum_{i-1}^{n-1}C_{i}S\right)}{A}, \end{array}$$

where *Q*_*t*_ is the cumulative amount of VitB3 permeated per unit area of the skin (*μ*g/cm^2^), *C*_*n*_ is the concentration of VitB3 measured at No. *n* sampling interval (*μ*g/ml), *C*_*i*_ is the concentration of VitB3 measured at No. *i* sampling interval (*μ*g/ml), *V* is the volume of individual Franz diffusion cell (ml), *S* is the volume of sampling aliquot, and *A* is the effective diffusion surface area, 1.77 cm^2^ [16, 23].

### 3.6. Deposition of HC-VitB3 Liposome in the Skin

After completing the in vitro permeation study (24 hours), the amount of VitB3 accumulated in the skin was analyzed [24]. The skin was removed from the diffusion cell, cut into small pieces, and put into a centrifuge tube containing 5 ml of methanol. Subsequently, it was vortexed for 2 minutes, sonicated for 15 minutes, and then centrifuged at 10,000 rpm (4°C for 10 minutes). Finally, the supernatant was analyzed for VitB3 content using UV spectrophotometer.

### 3.7. Preparation of HC-VitB3 Liposome Serum

HC-VitB3 liposome serum and HC-VitB3 serum were prepared by mixing all the ingredients in water. For preparation of the HC-VitB3 serum, each ingredient, such as disodium EDTA, HC, VitB3, polysorbate 20, propylene glycol, and phenoxyethanol was added to the water and continuously stirred until a clear solution was obtained. On the other hand, for preparing the HC-VitB3 liposome serum, the same preparation ingredients in same concentrations as in HC-VitB3 serum was added to a liposome suspension containing HC and VitB3. Then, sodium carboxymethyl cellulose 0.5–1.0% w/v was added to both solutions to increase the viscosity of the formulations [25].

### 3.8. Characterization of HC-VitB3 Liposome Serum

The physical properties of HC-VitB3 liposome serum and HC-VitB3 serum were assessed visually, including color and appearance, and photographs were taken. Color determination of both formulations was done using a CM 700d spectrophotometer (Konica Minolta, Inc., Tokyo, Japan). Total difference in color (Δ*E*^*∗*^) between the HC-VitB3 liposome serum and the HC-VitB3 serum was calculated using equation (1) [4]. pH of formulation was measured by a pH meter (Mettler Toledo pH510), and viscosity was measured by Brookfield viscometer DV-III ultrarheometer with a LV spindle. Both pH and viscosity were measured at 25°C in triplicate [25].

### 3.9. Product Satisfaction Assessment

This study received prior approval from the Ethics Committee of the Faculty of Pharmaceutical Sciences at Prince of Songkla University, Thailand (MHESI 68108/2722). Informed consent was obtained from all volunteers before their participation in the study. The satisfaction assessment was a comparative study using the same tester to test each product with a double-blind study. Therefore, equation (5) [26] was used to calculate the minimum number of participants to ensure reliable results for product satisfaction assessment. Inclusion criteria were as follows: the minimum number of volunteers required was at least 46 healthy male or female, who were aged between 21 and 70 years, willing to agree to the product assessment testing protocols, sign the informed consent, and did not have any skin disease. Exclusion criteria were as follows: having histories of allergies from natural ingredients or chemicals contained in the HC-VitB3 liposome serum and HC-VitB3 serum formulation [25]. Testing was conducted by having volunteers apply the product to the forearm area, assess the odor, and observe the various characteristics of both the products. The assessment included evaluating the satisfaction with regard to smell, color, texture, and skin absorption. The volunteers were not informed about which products were HC-VitB3 serum and HC-VitB3 liposome serum. The evaluation process took 30 minutes and utilized the abovementioned criteria to measure satisfaction levels, employing a Likert scale. The characteristics were determined using a 5-level rating scale, which includes the highest (5), high (4), moderate (3), low (2), and the lowest (1) levels of satisfaction. At the end of the evaluation, volunteers selected their preferred products and provided the reasons.

(5) $$\begin{array}{c} n={\left[\frac{\left(Z_{\alpha \;}+Z_{\beta \;}\;\right)\;\sigma \;}{d}\right]}^{2};\;\sigma =\sigma_{1}^{2}+\sigma_{2}^{2}\;–\;2r\sigma_{1}\sigma_{2}, \end{array}$$

where *n* is the required sample size, *Z*_*α* _ is 1.96 when *α* = 0.05, *Z*_*β* _ is 1.28 when *β* = 0.10, *σ* is standard deviation, *σ*_1_ is standard deviation of testing product 1, *σ*_2_ is standard deviation of testing product 2, *r* is 0 for getting the greatest number of sample size, and *d* is the difference in means of satisfaction score.

### 3.10. Statistical Analysis

The data were expressed as the mean ± SD, and the statistical analyses were conducted using either one-way ANOVA or a *t*-test, depending on the data evaluation. Color evaluation of HC-VitB3 liposomes and HC-VitB3 solution was analyzed using one-way ANOVA. Cell proliferation, migration of human fibroblast, soluble collagen production, skin permeation, and skin deposition of HC-VitB3 liposome were analyzed using *t*-test. Physicochemical properties and product satisfaction of HC-VitB3 liposome serum and HC-VitB3 serum were analyzed using *t*-test. The statistical differences between values showing *P* < 0.05 were considered as significant.

## 4. Results and Discussion

### 4.1. HC-VitB3 Liposome and Physicochemical Properties

Ten formulations of liposomes containing HC-VitB3 with varying ratios of SPC: CHOL: Tween 80/Span 80 were prepared. The selection criteria were based on physical characteristics, such as their ability to mask odors, and color of freshly prepared samples compared to HC-VitB3 solution samples as well as all samples after one week (Table 2). Once the suitable formulations were identified from Table 2, an evaluation was conducted to determine the color, particle size, polydispersity index (PDI), zeta potential, and percentage of entrapment efficiency in order to select the appropriate formulation.

The results shown in Table 2 revealed that the HC-VitB3 solution exhibited a clear gray color and emitted a very strong fishy odor when compared to all of the HC-VitB3 liposomes. However, HC-VitB3 liposomes F1 and F3, after being stored for one week, emitted a stronger fishy odor than their freshly prepared counterparts. Therefore, these two formulations were eliminated due to not meeting the selection criteria. Eight formulations of HC-VitB3 liposomes underwent assessment for particle size, polydispersity index (PDI), zeta potential, and percentage of entrapment efficiency, as displayed in Table 3. The color of these formulations is shown in Table 4.

All eight formulations of HC-VitB3 liposomes were nanosized ranging from 161 to 344 nm, exhibited low PDI values (0.1–0.3), and possessed zeta potential values ranging from −19 to −41 mV. The formulation with Tween 80 had a smaller particle size compared to the one with Span 80. All liposomes exhibited a negative value of zeta potential, primarily attributed to the negative charge of the phosphate group in phosphatidylcholine. Zeta potential serves as an indicator of the physical stability of liposomes [9]. The particle size tends to impact the charge distribution on the liposome surface [27]. Interestingly, each Span 80-based formula not only exhibited a larger size compared to the Tween 80 formula, but also featured a higher negative zeta potential value. This distribution of negative charges on the liposome surface results in repulsion between particles, reducing the likelihood of agglomeration or particle collision [9]. PDI is a measure of particle size distribution, indicating the variations in particle sizes within a sample. Liposomes with consistent particle sizes exhibit a low PDI [28]. Thus, favorable physical properties of liposomes include minimal particle size variation and a high zeta potential. In addition, a high percent entrapment efficiency is an essential characteristic of preferable liposome formulations. HC-VitB3 liposome formulations F2 and F4 exhibited the highest percent entrapment efficiency of VitB3 (95.31% and 95.73%, as shown in Table 3), which corresponds to their ability to mask the fishy odor, as indicated by the results in Table 2. The entrapment efficiency of HC in all eight formulations was in the range of 4.66–49.63%, which corresponds to the result of VitB3 entrapment. HC-VitB3 liposome formulation F4 gave the highest percent entrapment efficiency (49.63 ± 1.74%). However, the HC entrapment value in the F4 liposome formulation was still half of the VitB3 value. The efficiency of substance entrapment in liposomes is influenced by the proportions of various components. Liposomes contain cholesterol, which contributes to strengthening the liposome wall and maintaining its shape. However, when cholesterol levels surpass a specific threshold, they can disrupt the double-walled structure of liposomes, resulting in reduced substance entrapment efficiency [29]. Moreover, surfactants present in the liposome formula, such as Tween 80 or Span 80, have the potential to degrade the skin membrane and facilitate alterations in the shape of liposome bilayers, making them more suitable for their intended purpose [16]. The entrapment efficiency of the formulation is affected by the size of the active substance molecule, especially when comparing HC with a molecular weight (MW) of 102–10175 Da to VitB3 with a MW of 122 Da. Furthermore, the solubility of VitB3 in aqueous solutions is higher than that of HC, which allows liposomes to encapsulate VitB3 much more efficiently than HC. This corresponds to the findings of the review by Eloy et al. [30]. In Table 4, the color values of both the HC-VitB3 solution and HC-VitB3 liposomes reveal that the *L*^*∗*^ value of HC in every formula of liposomes was significantly higher than that of the HC-VitB3 solution (*P* < 0.05), indicating an increased brightness value for the HC-VitB3 liposomes. When calculating the total color difference value (∆*E*) by comparing it with the HC-VitB3 solution, it was observed that each HC-VitB3 liposome formulation had a significantly different ∆*E* value (*P* < 0.05). This indicates that the liposome preparation reduced the grayness of the collagen, with the external color being attributed to the phospholipids. The color value is influenced by the ingredients composing the liposomes [31].

**Figure 1:**
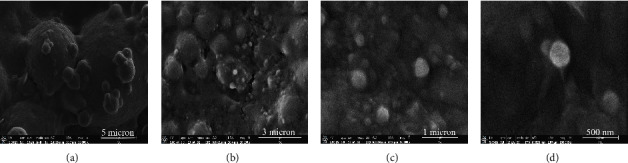
SEM photographs of HC-VitB3 liposome (F4) with magnification 10 k (a), 20 k (b), 50 k (c), and 100 k (d).

Based on the criteria for selecting HC-VitB3 liposomes, both F2 and F4 were the most effective formulations for encapsulating substances. When evaluating the color difference between HC-VitB3 liposomes and the HC-VitB3 solution, it was observed that formula F4 yielded the highest ∆*E* value, resulting in a bright yellow color formulation. Therefore, F4 was chosen as the optimal formulation for testing skin penetration and its effectiveness in masking the fishy odor when compared to the HC-VitB3 solution. Furthermore, morphology of HC-VitB3 liposome (F4) was determined by SEM is shown in Figure 1.

The morphology of HC-VitB3 liposomes, as revealed in SEM photographs, shows spherical vesicular structures. The size of the vesicles corresponded to the particle size measurement obtained from the zeta potential analyzer (170 nm).

### 4.2. Odorant of HC-VitB3 Liposome

**Figure 2:**
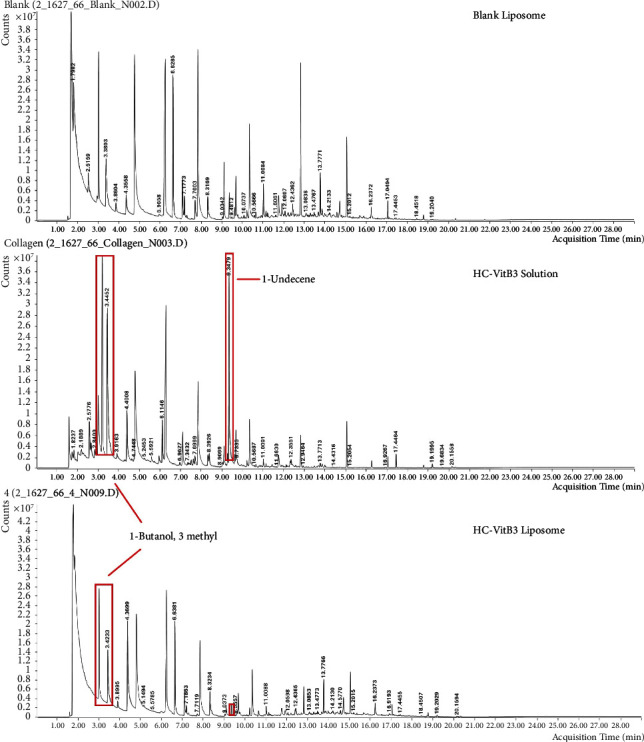
GC-MS chromatogram of blank liposome, HC-VitB3 solution, and HC-VitB3 liposome (F4).

The odor of three samples, blank liposome, HC-VitB3 solution, and HC-VitB3 liposome (F4) were analyzed by GC-MS technique. Chromatograms of three samples are shown in Figure 2. It was found that at 3.2026 minutes, 3.4233 minutes, and 9.3479 minutes, peaks were observed in the HC-VitB3 solution, but they were not present in the HC-VitB3 liposome. The peak at 3.4452 minutes was identified as 1-butanol, 3-methyl, a substance known for its unpleasant odor [32]. Likewise, the peak at 9.3479 minutes was identified as 1-undecene, which is also associated with an odor [33]. However, the substance responsible for the peak at 3.2026 minutes could not be identified. Based on these observations, it can be inferred that liposomal-hydrolyzed collagen is effective in reducing the odors caused by 1-butanol, 3-methyl, and 1-undecene present in the HC-VitB3 solution.

### 4.3. Cell Viability of HC-VitB3 Liposome

**Figure 3:**
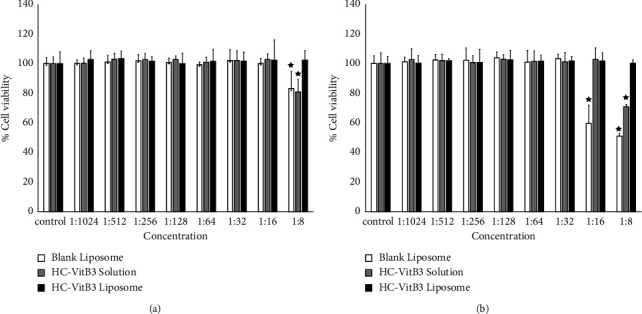
The percentage of cell viability for human skin fibroblasts (BJ, (a)) and human keratinocytes (HaCaT cells, (b)) was assessed when testing the cytotoxicity of blank liposomes, HC-VitB3 solution, and HC-VitB3 liposomes at various concentrations. Bars represent standard deviation (*n* = 3). ^*∗*^On bars indicate significant differences between different samples tested and control (*P* < 0.05).

The toxicity of blank liposome, HC-VitB3 solution, and HC-VitB3 liposome was assessed in BJ cells and HaCaT cells at concentrations ranging from 19.5 *μ*g/ml to 2.5 mg/ml. As depicted in Figure 3, it was observed that at every concentration ratio of HC-VitB3 liposome, the percentage cell survival values in BJ and HaCaT cells did not differ significantly from those in the control group. However, both the blank liposome and the HC-VitB3 solution exhibited significantly lower cell viability percentages compared to the control group. In the BJ cell test, at a concentration ratio of 1 : 8 (2.5 mg/ml), the percentage cell survival values of blank liposome and HC-VitB3 solution were 82.88 and 80.79, respectively. In the HaCaT cell test, the cell survival percentage of blank liposome significantly reduced to below 60 percent at concentration ratios ranging from 1 : 16 to 1 : 8 (1.25–2.5 mg/ml), whereas the survival percentage of HC-VitB3 solution decreased to 70.85 percent at a concentration of 2.5 mg/ml. Therefore, we can conclude that, in this study, HC-VitB3 liposome does not induce cell toxicity at any concentration.

### 4.4. Effect of HC-VitB3 Liposome on Migration of BJ and HaCaT Cells

**Figure 4:**
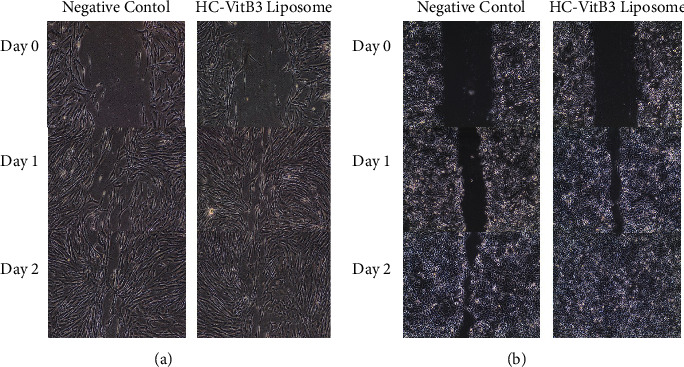
Measurement of cell migration in the in vitro scratch assay: (a) BJ and (b) HaCaT layer subjected to scratch and treated with HC-VitB3 liposome at days 0, 1, and 2 after incubation.

HC-VitB3 liposome affected the cell migration of BJ and HaCaT cells as shown in Figure 4. HC-VitB3 liposomes significantly stimulated cell migration compared to the negative control. The percentage of migration in BJ cells treated with HC-VitB3 liposomes was notably higher than that in the negative control for both cell lines. Specifically, on day 1, a concentration of 2.5 mg/ml of HC-VitB3 liposomes increased BJ cell migration to 74.80 ± 0.89%, while the negative control exhibited a migration of only 35.24 ± 0.97%. Moreover, a concentration of 2.5 mg/ml of HC-VitB3 liposomes enhanced HaCaT cell migration to 65.94 ± 6.53% compared to the negative control's 45.65 ± 3.90% on day 1. Consequently, HC-VitB3 liposomes demonstrated superior wound healing efficacy compared to the control group (*P* < 0.05). These results confirmed the effective wound healing activity of HC-VitB3 liposome formulation [5].

### 4.5. Soluble Collagen Production of HC-VitB3 Liposome

Blank liposomes and HC-VitB3 liposomes in various concentrations were used to stimulate collagen production in the BJ cell line and compared to the results of the control. At a concentration of 1.25 mg/ml, only HC-VitB3 liposomes significantly increased collagen production in BJ cells, resulting in significantly higher amounts (40.92 ± 25.87 *μ*g/ml) compared to the control (11.34 ± 1.74 *μ*g/ml). Furthermore, when the concentration was increased to 2.5 mg/ml, soluble collagen production reached 256.61 ± 25.89 *μ*g/ml. In contrast, blank liposome did not stimulate collagen production in BJ cells. These results revealed that HC-VitB3 liposome exhibited good collagen production activity and was nontoxic to both cells. In addition, HC-VitB3 liposome promoted collagen production in BJ cells in a concentration-dependent manner similar to *Jatropha curcas* L extract [22].

### 4.6. Skin Permeation and Skin Deposition of HC-VitB3 Liposome

**Figure 5:**
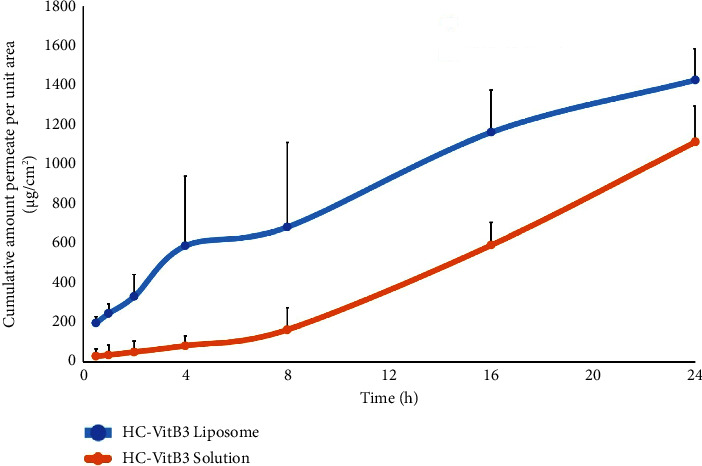
Cumulative amount of permeation of HC-VitB3 solution and HC-VitB3 liposome (mean ± SD, *n* = 4).

The skin permeation of HC-VitB3 liposome through pig ear skin was evaluated. The physiological, biochemical, and histological characteristics of porcine ear skin exhibit many similarities when compared to human skin in terms of lipid position and thickness [34, 35]. The in vitro skin permeation profiles of HC-VitB3 solution and HC-VitB3 liposome are shown in Figure 5. Among the formulations studied, formulation F4 proved to be the most effective HC-VitB3 liposome in skin penetration when compared to HC-VitB3 solutions. In the experiment, VitB3 served as a representative compound to assess the entrapment and skin penetration of liposomes. Both HC and VitB3 are water-soluble substances and are located within the central cavity of the liposome vesicles. However, the entrapment efficiency of HC in F4 liposome was only 49.63% which was lower than VitB3 (95.73%). It was observed that HC-VitB3 liposomes exhibited a significantly higher accumulation of VitB3 in the receptor fluid at 0.5, 1, 2, 4, 8, 16, and 24 hours, in contrast to the HC-VitB3 solution (*P* < 0.05). This suggests that HC and VitB3 encapsulated within liposome vesicles can readily traverse the layers of the skin and accumulate in both the epidermis and dermis. This capability is due to the lipid bilayer structure, which closely resembles the skin's cell membrane, enabling liposomes to penetrate the cell membrane space and deliver encapsulated substances to their target locations. Moreover, liposomes contain surfactants, such as Tween 80, which can alter the fluidity of the cell membrane. This alteration causes denaturation of the cell membrane wall [13], facilitating the enhanced permeation of liposomes into the intercellular spaces. The formulations of liposomes thus prove advantageous in promoting the absorption of various substances through the skin. The low permeability of all compounds might be due to the structure of the outermost skin layer, the stratum corneum. It consists of protein-rich corneocytes embedded in a matrix of lipids. This domain is the rate-limiting step for the penetration of drugs [36]. Analysis of the accumulated amount of VitB3 in the skin revealed that HC-VitB3 liposomes exhibited a value of 1442.67 ± 106.17 *μ*g of VitB3 accumulation in pig skin, which was significantly higher than the 1003.37 ± 89.20 *μ*g observed in the HC-VitB3 solution (*P* < 0.05). These results, consistent with numerous studies [13–15], indicate that liposomes employ various mechanisms to facilitate skin absorption, enabling substances to penetrate deeper into the skin.

### 4.7. HC-VitB3 Liposome Serum and Physicochemical Properties

**Figure 6:**
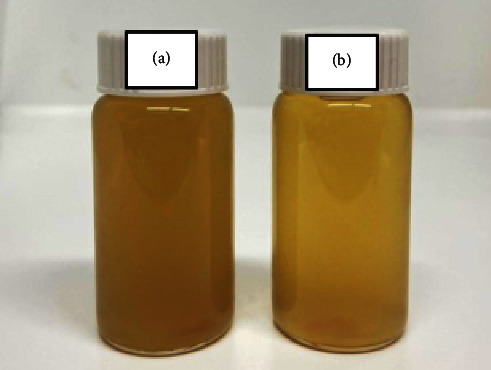
HC-VitB3 serum (a) and HC-VitB3 liposome serum (b).

HC-VitB3 liposome serum was a clear and a bright yellow solution compared to HC-VitB3 serum, as shown in Figure 6. The evaluation of physical properties included color, pH measurement, and viscosity measurement of both formulations and is shown in Table 5. The results of the colorimetric test indicated that the HC-VitB3 liposome serum product exhibited a brighter appearance, as evidenced by a significantly higher *L*^*∗*^ value (*P* < 0.05), and more pronounced red and yellow hues. Moreover, both the *a*^*∗*^ and *b*^*∗*^ values were significantly greater (*P* < 0.05) for the liposomal formulation, which can be attributed to the inherent yellow color of soybeans. Notably, the presence of phosphatidylcholine in the liposome walls [37] contributed to a color difference value of 3.05, when compared to the HC-VitB3 serum. The pH value of the HC-VitB3 serum was 6.94, whereas the pH value of the HC-VitB3 liposome serum was 7.04, indicating a slightly more alkaline nature. This difference can be attributed to the presence of liposome components. Regarding viscosity, the HC-VitB3 serum had a viscosity of 2,913.33 cPs, while the HC-VitB3 liposome serum exhibited a higher viscosity of 5,400 cPs. This increased viscosity is primarily due to the presence of lipids, such as phosphatidylcholine from soybeans and cholesterol. The viscosity of the serum formulation plays an important role on deciding whether it feels comfortable or not when applied on the skin; furthermore, viscosity depends on the amount of thickening agent [25]. In this study, the viscosity of the HC-VitB3 liposome serum was higher than that of the HC-VitB3 serum by almost two times. However, the results showed that the skin permeation value of the liposome was still higher than that of the solution. A balance between viscosity and absorptivity on the skin is one of the many factors that influence the satisfaction of the product users. Furthermore, the HC-VitB3 liposome serum followed the quality critical attributes such as physical quality and stability of the product as stated in design and development of topical liposomal formulations in a regulatory perspective [38].

### 4.8. Satisfaction Assessment of HC-VitB3 Liposome Serum

A satisfaction test was conducted with 100 volunteers aged 18–28 years, comprising 24 males and 76 females, to assess their satisfaction regarding the color, smell, texture, and absorption of the HC-VitB3 liposome serum and HC-VitB3 serum, each scored on a scale of 5. The results are presented in Table 6. Satisfaction scores for texture and absorption did not exhibit significant differences (*P* > 0.05) because some volunteers preferred the texture of the HC-VitB3 serum, finding it lighter and less heavy on the skin. Conversely, some volunteers favored the texture of the HC-VitB3 liposome serum because it provided more moisture after application, resulting in no significant difference in texture scores. However, satisfaction with the color of the HC-VitB3 liposome serum product was significantly lower than that of the HC-VitB3 serum (*P* < 0.05). This was because volunteers were not shown the color of the serum stored in an opaque bottle. The color of the serum became apparent only when a small amount was dispensed, leading most volunteers to rate their satisfaction with the color of the HC-VitB3 serum higher than that of HC-VitB3 liposome serum. In terms of scent, satisfaction of volunteers with the HC-VitB3 liposome serum product was significantly higher than that of HC-VitB3 serum (*P* < 0.05). This was due to HC-VitB3 liposome effectively masking any fishy odors, allowing the perfume scent to become more prominent. In contrast, the HC-VitB3 serum had a noticeable fishy smell, and the applied perfume could not effectively mask it, resulting in an unpleasant scent. All the 100 volunteers reported no irritation after using both the HC-VitB3 serum and HC-VitB3 liposome serum. When volunteers were asked to evaluate the products and select their preferred option, 73 of them chose the HC-VitB3 liposome serum. Most of them cited the scent as a key factor influencing their choice. On the other hand, 27 people chose the HC-VitB3 serum, appreciating its lighter texture and being less sensitive to the scent. Interestingly, most males chose the HC-VitB3 serum, which is consistent with research showing that males' ability to discriminate different odors is poorer than females [39]. Some volunteers expressed the opinion that the HC product derived from fish should have a slightly fishy odor due to certain active substances present in the product. However, the product development concept prioritizes quality and acceptability, so the product should remain stable for a specified period. Therefore, liposomes were used to incorporate HC for masking odor and enhancing the quality of the cosmetic product.

### 4.9. Potential Cosmetic Applications of HC-VitB3 Liposome Serum

From results of all the studies, the developed liposome with a proper lipid bilayer structure and ratio has the potential to offer both effective concealment of fishy odors and unpleasant colors, as well as high skin penetration. Furthermore, HC-VitB3 liposome did not induce cell toxicity and exhibited good collagen production and effective wound healing activity. The liposome serum was prepared using a concentration of effective ingredients. The HC-VitB3 liposome serum represents an innovative approach in the field of cosmetic skincare. Hydrolyzed collagen, a key component of the serum, is known for its ability to replenish and retain moisture in the skin. When combined with VitB3, it forms a potent hydrating complex that penetrates deep into the skin's layers, promoting optimal hydration levels. This results in improved skin elasticity and firmness, reducing the appearance of fine lines and wrinkles [14, 25]. The combination of HC, VitB3, and liposomes holds immense potential for various cosmetic applications. From enhancing skin hydration and firmness to promoting collagen synthesis and brightening the complexion, this innovative liposome serum offers multifaceted benefits for skin health and rejuvenation. Further research and clinical studies could highlight its efficacy and establish its position as a valuable product in cosmetic skincare regimens.

## 5. Conclusion

HC-VitB3 liposomes can improve the physicochemical properties and reduce the odor and undesirable color of the extract while enhancing absorption through the skin. Utilizing a suitable weight ratio of phosphatidylcholine, cholesterol, and Tween 80 at 8 : 2 : 1 results in the production of nanosized vesicles, which is promising for the development of advanced cosmetic products. The HC-VitB3 liposome serum also effectively masks fishy odors. Furthermore, none of the 100 volunteers reported experiencing irritation after using both the HC-VitB3 serum and HC-VitB3 liposome serum. In the overall evaluation, a majority of the volunteers selected HC-VitB3 liposome serum as their preferred product. Their rationale lies in the significant impact of scent on the usability of cosmetic products. Thus, it can be concluded that the development of this liposome product elevates the value of HC extracted from salmon fish (which would otherwise be considered a waste) by turning it into a valuable ingredient in the beauty industry.

## Figures and Tables

**Table 1:** The compositions of HC-VitB3 liposome formulations.

Formula	Compositions	Weight ratio	Total lipid (*μ*g/ml)
F1	SPC: CHO: Tween 80	8 : 1 : 1	31.43
F2	SPC: CHO: Tween 80	8 : 1 : 2	31.43
F3	SPC: CHO: Tween 80	8 : 1 : 3	31.43
F4	SPC: CHO: Tween 80	8 : 2 : 1	34.92
F5	SPC: CHO: Tween 80	8 : 3 : 1	38.42
F6	SPC: CHO: Span 80	8 : 1 : 1	31.43
F7	SPC: CHO: Span 80	8 : 1 : 2	31.43
F8	SPC: CHO: Span 80	8 : 1 : 3	31.43
F9	SPC: CHO: Span 80	8 : 2 : 1	34.92
F10	SPC: CHO: Span 80	8 : 3 : 1	38.42

**Table 2:** Physical properties of HC-VitB3 liposomes in terms of odor and color comparing to HC-VitB3 solution.

Formula	Freshly prepared		One week stored	
	Color	Fishy odor	Color	Fishy odor
F1	Clear brown	++++	Clear brown	+++++
F2	Turbid yellow	++	Turbid yellow	++
F3	Turbid yellow	+++	Turbid yellow	++++
F4	Turbid yellow	++	Turbid yellow	++
F5	Turbid yellow	++	Turbid yellow	+++
F6	Turbid white	++	Turbid white	+++
F7	Turbid white	++	Turbid white	+++
F8	Turbid white	++	Turbid white	+++
F9	Turbid white	++	Turbid white	+++
F10	Turbid white	++	Turbid white	+++
HC-VitB3 solution	Clear gray	+++++	Clear gray	+++++

A 5-level rating scale, which includes the highest (+++++), high (++++), moderate (+++), low (++), and the lowest (+) levels of smell of fishy odor.

**Table 3:** Particle size, polydispersity index (PDI), zeta potential, and percentage of entrapment efficiency of HC-VitB3 liposomes.

Formula	Size (nm)	PDI	Zeta potential (mV)	%EEVitB3
F2	168.8 ± 0.9	0.290 ± 0.005	−21.80 ± 1.14	95.31 ± 0.01
F4	170.6 ± 0.7	0.161 ± 0.008	−19.32 ± 0.72	95.73 ± 2.00
F5	160.7 ± 1.3	0.098 ± 0.012	−20.62 ± 0.71	10.22 ± 0.01
F6	223.7 ± 1.5	0.153 ± 0.027	−32.57 ± 0.92	27.12 ± 0.01
F7	247.3 ± 4.8	0.114 ± 0.031	−41.36 ± 0.71	19.57 ± 0.01
F8	343.5 ± 2.7	0.097 ± 0.028	−37.52 ± 1.11	8.98 ± 0.02
F9	193.6 ± 7.2	0.185 ± 0.025	−25.85 ± 0.59	16.22 ± 0.01
F10	194.0 ± 3.5	0.201 ± 0.014	−34.00 ± 1.35	24.32 ± 0.01

Data are expressed as mean ± SD (*n* = 3).

**Table 4:** The color of HC-VitB3 liposomes and HC-VitB3 solution.

Formula	*L* ^*∗*^	*a* ^*∗*^	*b* ^*∗*^	∆*E*
F2	35.08 ± 0.00^i^	−1.02 ± 0.01^e^	−3.54 ± 0.01^d^	8.63 ± 0.01^a^
F4	38.51 ± 0.00^h^	−1.12 ± 0.01^d^	−0.62 ± 0.01^a^	11.54 ± 0.00^b^
F5	41.95 ± 0.000^g^	−1.82 ± 0.00^g^	−1.49 ± 0.01^c^	15.06 ± 0.00^c^
F6	39.87 ± 0.00^f^	−1.04 ± 0.01^e^	−1.53 ± 0.01^c^	12.90 ± 0.00^d^
F7	43.58 ± 0.00^e^	−0.64 ± 0.01^f^	−0.39 ± 0.01^e^	16.54 ± 0.00^e^
F8	50.74 ± 0.00^d^	−1.09 ± 0.01^d^	2.88 ± 0.01^f^	23.97 ± 0.00^f^
F9	42.59 ± 0.00^c^	−1.25 ± 0.01^c^	0.03 ± 0.01^g^	15.62 ± 0.00^g^
F10	42.48 ± 0.00^b^	−1.43 ± 0.00^b^	−1.70 ± 0.01^b^	15.55 ± 0.00^h^
HC solution	27.08 ± 0.87^a^	0.48 ± 0.07^a^	−0.66 ± 0.36^a^	—

Data are expressed as mean ± SD (*n* = 3). Significance values (*P* < 0.05) are shown with different letters in each column.

**Table 5:** Color value, pH, viscosity of HC-VitB3 liposome serum, and HC-VitB3 serum.

Formula	*L* ^*∗*^	*a* ^*∗*^	*b* ^*∗*^	∆*E*	pH	Viscosity (cPs)
HC-VitB3 liposome serum	24.18 ± 0.00^b^	1.81 ± 0.01^b^	2.17 ± 0.01^b^	3.05 ± 0.01	7.04 ± 0.02^b^	5400.00 ± 57.74^b^
HC-VitB3 serum	21.66 ± 0.01^a^	0.86 ± 0.00^a^	1.40 ± 0.01^a^	—	6.94 ± 0.01^a^	2913.33 ± 59.26^a^

Data are expressed as mean ± SD (*n* = 3). Significance values (*P* < 0.05) are shown in different letters in the same column.

**Table 6:** Results of satisfaction assessment among 100 volunteers.

Topic	Satisfaction scores	
	HC-VitB3 serum	HC-VitB3 liposome serum
Color	4.28 ± 0.70^a^	3.92 ± 0.85^b^
Odor	2.44 ± 0.86^a^	3.57 ± 0.84^b^
Texture	4.31 ± 0.66^a^	4.26 ± 0.80^a^
Absorption	3.82 ± 0.80^a^	3.83 ± 0.82^a^

Data are expressed as mean ± SD (*n* = 100). Significance values (*P* < 0.05) are shown in different letters in the same row.

## Data Availability

No data were used to support the findings of this study.

## Additional Points

*Highlights*. (i) Liposomes-encapsulating hydrolyzed salmon collagen effectively enhance the concealment of unpleasant odors and colors. (ii) Liposomes enhance skin permeation of active ingredients. (iii) Hydrolyzed salmon collagen encapsulated in liposome offers a novel, innovative, and advanced approach in delivering cosmetic products with superior quality and effectiveness. (iv) The proper lipid bilayer structure of liposomes has the potential to offer both effective concealment of undesirable odors and colors, as well as high skin penetration.
